# Intraretinal microvascular changes after ERM and ILM peeling using SSOCTA

**DOI:** 10.1371/journal.pone.0242667

**Published:** 2020-12-01

**Authors:** Reinhard Told, Michael Georgopoulos, Gregor Sebastian Reiter, Lorenz Wassermann, Leyla Aliyeva, Lukas Baumann, Claudette Abela-Formanek, Andreas Pollreisz, Ursula Schmidt-Erfurth, Stefan Sacu

**Affiliations:** 1 Department of Ophthalmology and Optometry, Vienna Trial Center (VTC), Medical University of Vienna, Vienna, Austria; 2 Department of Medical Statistics Vienna, Medical University of Vienna, Vienna, Austria; University of Florida, UNITED STATES

## Abstract

**Background:**

To prospectively investigate retinal vascular changes in patients undergoing epiretinal membrane (ERM) and internal limiting membrane (ILM) peeling using swept source optical coherence tomography angiography (SSOCTA).

**Methods:**

Consecutive patients were grouped based on ERM severity and followed using SSOCTA up to month 3 after surgical intervention. Superficial and deep foveal avascular zone (s/dFAZ) as well as foveal and parafoveal vessel density (VD) were correlated with ERM severity and visual acuity. Differences between groups were evaluated.

**Results:**

Significant correlations were found between ERM severity and baseline sFAZ, dFAZ and best corrected visual acuity (BCVA), central retinal subfield thickness (CST) and ΔCST (r = -0.52, r = -0.43, r = -0.42, r = 0.58, r = 0.39; all p<0.05). Vascular flow parameters did not correlate with age, peeling size, pseudophakia or CST, but correlated with intraretinal cysts presence. No associations of BCVA with any of the OCTA parameters across time were found.

Significant differences between ERM severity groups 1 and 2 were found for sFAZ at baseline (p = 0.005) and at the 3-month follow-up (p = 0.014), and for dFAZ at baseline (p = 0.017). Superficial foveal and parafoveal VD were not significantly different between groups (all p>0.05).

**Conclusions:**

This study clearly shows that ERM severity based on ERM staging has to be taken into account when undertaking studies in patients with idiopathic ERM using SSOCTA. Further, specific changes in the superficial and deep retinal vasculature in eyes undergoing ERM and ILM peeling were found. However, the clinical usefulness and prognostic value for post-surgical treatment BCVA of the SSOCTA-derived variables (sFAZ and dFAZ area, as well as foveal and parafoveal VD) used remains questionable.

## Introduction

Idiopathic epiretinal membrane (ERM) is one of the most common macular diseases in the elderly with an incidence of 4–6%, increasing with age [1–4]. It causes metamorphopsia and visual acuity decreases slowly with disease severity [5]. Proliferative cells migrating along the surface of the internal limiting membrane (ILM) cause wrinkling and consequently retinal traction in the macular area [6]. Light and electron microscopic analyses suggest that histologically various cell types, such as glial cells, hyalocytes, retinal pigment epithelium cells and fibroblast-like cells, play a key role in ERM formation. The exact mechanism however, is still unknown [7].

The most frequent surgical approach in patients with symptomatic ERM is pars plana vitrectomy, followed by peeling of the membrane and the ILM aimed at restoring normal retinal structure and function [8,9]. Despite structural restoration after ERM and ILM peeling, symptoms do continue to persist in some patients. Therefore, identifying indicators for postoperative visual outcome is an urgent quest.

A myriad of morphologic and functional markers have been studied in an attempt to predict postoperative outcome. [6, 10–14] So far, preoperative visual acuity is the only variable consistently associated with postoperative best corrected visual acuity (BCVA) [14]. Amongst others the severity of metamorphopsia, inner segment/outer segment integrity on optical coherence tomography (OCT) and cone outer segment tip integrity have been shown to be possibly associated with postoperative BCVA [14–18]. An ERM staging system was introduced based on the association of the thickness of the inner retinal layers with vision loss in ERM eyes [10].

Fluorescein angiography was used in previous studies to assess the foveal avascular zone (FAZ) area. Perifoveal leakage and decreased blood flow [19], with partial improvement after ERM peeling has been demonstrated [20], indicating a hemodynamic component in the pathophysiology. With the availability of optical coherence tomography angiography (OCTA) it is now possible to non-invasively image the different vascular plexuses of the retina in high resolution [21,22]. The purpose of this study was to prospectively evaluate OCTA-based variables in patients undergoing ERM and ILM peeling amd further, to determine the influence of ERM severity on OCTA parameters and correlate OCTA variables with function as well as morphologic OCT parameters. The dynamics of the FAZ area of the superficial and deep retinal plexuses, a factor reported potentially to predict visual acuity outcome following surgical intervention [23,24], were considered and additional factors including vessel density (VD), peeling area and specific retinal abnormalities evaluated.

## Materials and methods

This prospective, clinical study was approved by the Ethics Committee of the Medical University of Vienna (EK1020/2014) and followed the tenets of the Declaration of Helsinki. All participants signed an informed consent form after receiving an in-depth explanation of the study and before being included in the study.

The consecutive patients included had to be over 18 years of age and have an idiopathic epiretinal membrane scheduled to undergo pars plana vitrectomy and ERM and ILM peeling without endotamponade. Patients with cataract underwent a combined procedure with phacoemulsification and intraocular lens implantation. Exclusion criteria were any other eye disease or abnormalities that prevented reliable measurements, e.g. severe cataract or ametropia exceeding +/- 3 diopters spherical equivalent. Consequently, an image quality score below 40 was also considered an exclusion criterion [25]. In case of previous cataract surgery, only patients with bilateral pseudophakia at baseline were eligible for study inclusion. Patients with systemic disease such as diabetes mellitus or who had participated in a clinical trial in the 3 weeks preceding the study start were excluded from this study. The healthy contralateral eye was used as the control.

All patients underwent a comprehensive ophthalmic examination, including BCVA testing using ETDRS charts [26], intraocular pressure (IOP) assessment using a Goldmann applanation tonometer, slit-lamp examination, and swept source (SS) OCTA (DRI OCT Triton Plus, Topcon, Japan) as well as spectral domain (SD) OCT (Spectralis HRA + OCT, Heidelberg Engineering, Heidelberg, Germany) assessments. At each visit (baseline, day 1, week 1, month 1 and month 3), we performed an ophthalmic examination, applanation tonometry, slit-lamp examination, SSOCTA and SDOCT.

### Spectral domain optical coherence tomography

A cube comprising the central 6 x 6 mm of the posterior pole with a resolution of 1,024 x 49 (A-scans x B-scans) centered on the fovea was acquired. We ensured that the same retinal area was scanned at each visit by using the follow-up mode of the device. The software automatically calculated the central retinal subfield thickness (CST) within the central millimeter. Care was taken that the automated segmentations were correct and manual adjustments made if needed.

### ERM staging

ERM was staged based on OCT B-scans as described [10]. In short; Stage 1 is defined as a mild ERM with the foveal pit present and all retinal layers clearly identifiable outside the fovea, negligible morphologic or anatomic disruption; Stage 2 is defined as an ERM with associated retinal distortion including loss of the foveal depression and stretching of the outer nuclear layer, yet all retinal layers clearly identifiable outside the fovea; Stage 3 is defined as an ERM with the foveal depression absent, continuous ectopic inner foveal layer anomaly crossing the central foveal area, yet all retinal layers clearly identifiable; Stage 4 is defined as an ERM with anatomic disruption of the macula and pronounced retinal thickening, ectopic inner foveal layers crossing the entire fovea and retinal layers distorted and disorganized on OCT. Patients were grouped based on ERM severity: Group 1 comprised ERM stages 1 and 2, and Group 2 ERM stages 3 and 4.

### Optical coherence tomography angiography

The SSOCTA en face images obtained for the superficial and deep FAZ covered an area of 3x3mm and those for the VD 6x6mm. The built-in software (IMAGEnet6, v1.22.1.14101) was used to delineate the superficial capillary plexus (SCP) and the deep capillary plexus (DCP). The DCP delineated by this software was 15.6 μm below the junction between the inner plexiform and inner nuclear layer (IPL/INL) to 70.2 μm below the IPL/INL. Segmentations were corrected manually if necessary. The FAZ area was delineated manually using the software included on the device. VD evaluations were also performed with the system’s own software and exported based on the ETDRS grid. Consequently, we report foveal VD as VD1 and parafoveal VD as the mean of the ETDRS segments 2 to 5 (VD2-5), representing the inner ETDRS ring [27]. This approach was chosen as in the case of minimum decentration the 6x6-mm OCTA cube prevents data loss, which can occur in a 3x3-mm OCTA cube.

### Surgical procedure

All affected eyes underwent 23-gauge pars plana vitrectomy with ERM and ILM peeling under general anesthesia. Expert retinal surgeons (SS, MG and CA) at the Department of Ophthalmology and Optometry, Medical University of Vienna, Austria performed the operations. In short, a 3-port pars plana vitrectomy with ERM and ILM peeling after staining with View ILM (0.03% Blulife, Alchimia srl, Ponte San Nicolò, Italy) was performed. A combined procedure of ERM and ILM peeling with phacoemulsification and intraocular lens implantation was chosen for eyes with cataracts. The optic nerve head size was used as a measure to record the size of the peeling area. Standardized post-operative treatment comprised gentamycin with dexamethasone eye drops (Dexagenta-POS eye drops) as well as gentamycin with dexamethasone ointment (Dexagenta-POS ointment, both from Ursapharm Ges.m.b.H., Klosterneuburg, Austria) for the night.

### Statistical analyses

Statistical analyses were performed using SPSS version 22.0 (IBM Corporation, Armonk, NY, USA). Descriptive analyses are reported as the mean ± standard deviation (SD). BCVA was converted to the logarithm of the minimum angle of resolution (logMAR) equivalents for statistical evaluation. The Shapiro-Wilk test was used to test for normal distribution. A repeated measurements ANOVA was used to test for statistically significant differences over time. Each timepoint was tested with a post-hoc analysis using the least significant difference (LSD) test for the correction of multiple testing. We calculated Pearson correlation coefficients to assess associations between ERM severity and morphologic and vascular OCT/A variables. The point biserial correlation coefficient was calculated for correlations with dichotomous variables. A p-value of less than 0.05 was accepted as statistically significant.

## Results

After excluding patients whose images were of insufficient quality (N = 2) and patients lost in follow-up (N = 3), 32 eyes of 32 patients were included in this study. The mean age was 71 ± 7 years, 45.6% were women.

At baseline 21 patients were phakic and 11 pseudophakic. Fourteen eyes received an ERM and ILM peeling combined with phacoemulsification and intraocular lens implantation. Baseline characteristics and data grouped based on clinical severity stage are summarized in Table 1.

**Table 1:** Baseline characteristics of the superficial and deep FAZ area (sFAZ, dFAZ), para- and perifoveal vessel density (VD1; VD2-5), best corrected visual acuity (BCVA) and central subfield thickness (CST).

		Overall n = 32	Group 1 n = 18	Group 2 n = 14	p-value
**ERM stage (n)**	ERM eyes		Stage 1: n = 4	Stage 3: n = 11	
			Stage 2: n = 14	Stage 4: n = 3	
**sFAZ area (μm**^**2**^**)**	ERM eyes	114 ± 81	151 ± 89	65 ± 26	**0.005**
	control eyes	231 ± 114	223 ± 127	242 ± 99	
**dFAZ area (μm**^**2**^**)**	ERM eyes	168 ± 134	220 ± 147	97 ± 69	**0.017**
	control eyes	312 ± 130	315 ± 158	307 ± 90	
**VD 1 (%)**	ERM eyes	30.5 ± 21.4	29.5 ± 5.9	31.8 ± 6.0	0.340
	control eyes	21.4 ± 5.1	22.9 ± 5.6	19.5 ± 3.9	
**VD2-5 (%)**	ERM eyes	47.7 ± 3.2	47.9 ± 3.7	47.4 ± 2.7	0.690
	control eyes	47.0 ± 2.9	47.6 ± 3.0	46.4 ± 2.9	
**BCVA (ETDRS letters)**	ERM eyes	73 ± 11	76 ± 8	69 ± 13	0.084
	control eyes	81 ± 11	80 ± 13	83 ± 5	
**CST (μm)**	ERM eyes	459 ± 64	413 ± 63	510 ± 77	**0.001**
	control eyes	280 ± 54	278 ± 46	283 ± 63	
**Pseudophakia (n)**	ERM eyes	11	6	5	
	control eyes	11	6	5	

Data are presented as the mean ± SD; Data presented are grouped based on severity; Group 1 comprises ERM stages one and two, Group 2 comprises ERM stages three and four. P-value indicates changes between Groups 1 and 2 in ERM eyes.

### Foveal avascular zone (FAZ)

**Fig 1:**
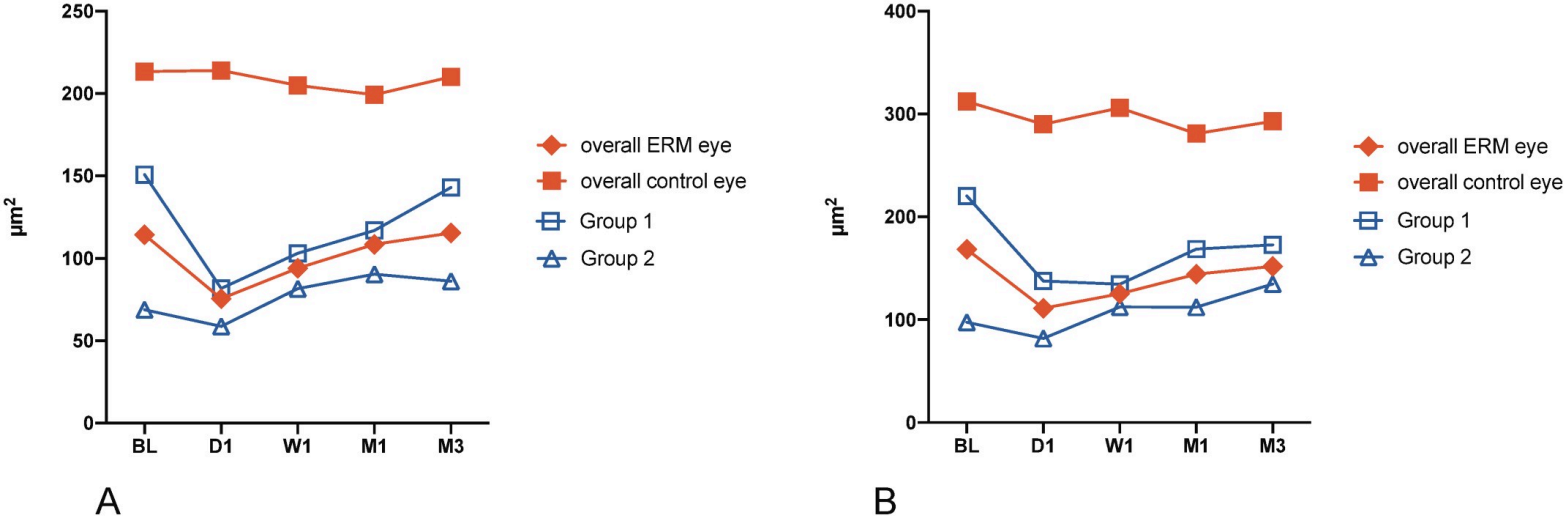
FAZ area of the (A) superficial and (B) deep retinal capillary plexus during the first 3 months after ERM + ILM peeling. Data are presented as the mean; BL = baseline, W1 = week 1, M1 = month 1, M3 = month 3, Group 1 = ERM stages 1 and 2; Group 2 = ERM stages 3 and 4.

The mean superficial retinal plexus FAZ was statistically significantly reduced at day 1 after surgery (p = 0.043), whereas the change in mean deep retinal plexus FAZ did not reach the level of significance (p = 0.056; see Fig 1A and 1B). Both, sFAZ and dFAZ increased slowly thereafter, reaching pre-surgical intervention values. There was no statistically significant difference from week 1 onwards, either in the superficial or deep retinal plexus. In healthy control eyes, there was no statistically significant change in either the superficial or deep FAZ between baseline and month one as well as at month 3 (all p>0.05).

Compared with healthy control eyes, the baseline mean FAZ of the superficial and deep retinal plexus were statistically significantly smaller in ERM eyes (p = 0.0002, p < 0.0001, respectively, see Table 1). This difference continued throughout month 3 (see Fig 1A and 1B).

Grouping patients based on ERM severity (see Fig 1A and 1B) showed that the superficial and deep FAZ both decreased in size with increasing ERM severity. This finding persisted at the 3-month follow-up. There were statistically significant differences in the superficial and deep FAZ between Groups 1 and 2 at baseline (p = 0.005; p = 0.017, respectively). There was still a statistically significant difference between Groups 1 and 2 in the superficial FAZ (p = 0.014) at the 3-month follow-up but not in the deep capillary plexus FAZ (p = 0.40).

### Vessel density (VD)

**Fig 2:**
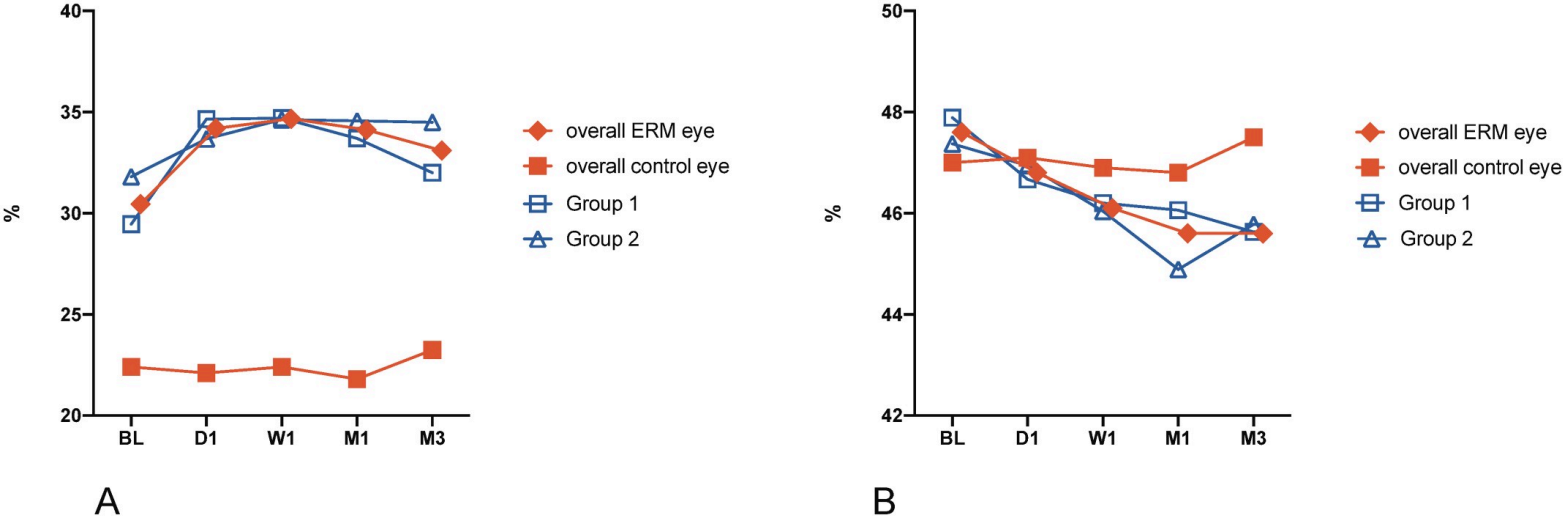
Vessel density (VD) of the superficial retinal plexus was assessed in ETDRS grid locations 1 to 5. (A) VD of the foveal and centermost ETDRS location 1 (VD1); (B) Mean VD of the parafoveal and surrounding ETDRS subfields 2–5 (VD2-5), representing the inner circle of the ETDRS grid. Data are presented as mean; BL = baseline, W1 = week 1, M1 = month 1, M3 = month 3; Group 1 = ERM stages 1 and 2; Group 2 = ERM stages 3 and 4.

There was a statistically significant difference in VD1 between ERM eyes and contralateral control eyes at baseline (p<0.0001, see Table 1). VD1 in ERM eyes changed significantly during the first 3 months after ERM peeling surgery (p = 0.0021; see Fig 2A), whereas, there was no statistically significant change in contralateral control eyes (p = 0.82).

At baseline, there was no statistically significant difference between ERM eyes and contralateral control eyes in VD2-5 (p = 0.92). The mean VD2-5 showed a statistically significant change during the first 3 months after surgical treatment (p < 0.001; see Fig 2B). Contralateral control eyes showed no statistically significant change during the 3-month observation period (p = 0.59).

Although both the VD1 and surrounding mean VD2-5 showed a statistically significant change during the observation period, their presentation after surgical intervention was different. VD1 increased by 9.9% after the first week and gradually decreased thereafter (Fig 2A), whereas mean VD2-5 decreased by 4.6% until month one and remained stable up to month 3 (see Fig 2B).

Grouping patients based on disease severity showed that there was no significant difference between groups either at baseline or 3 months after ERM + ILM peeling in VD1 (p = 0.34; p = 0.16, respectively) as well as VD2-5 (p = 0.69; p = 0.9, respectively). Consequently, the foveal and parafoveal VD of Groups 1 and 2 showed an almost identical course (see Fig 2A and 2B).

### Correlations

**Fig 3:**
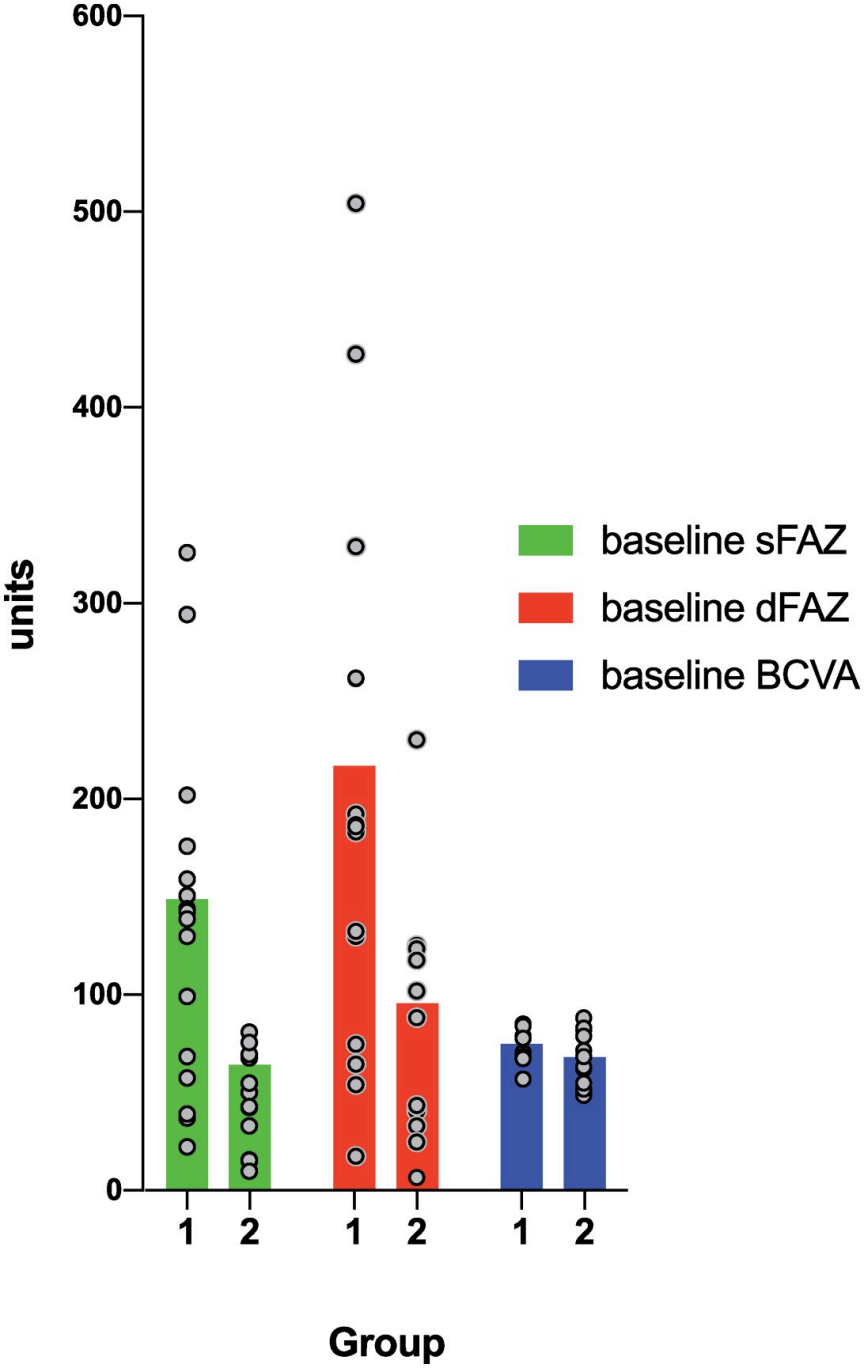
Parameters correlating with ERM severity. Data shows mean baseline sFAZ area, dFAZ area, BCVA, which show significant correlations with ERM severity (Group 1 = ERM stages 1 and 2; Group 2 = ERM stages 3 and 4). The y-axis units refer to the appropriate measures of the before-mentioned variables (sFAZ and dFAZ area: μm^2^, BCVA: ETDRS letters). Grey dots represent individual values; note that some lay on top of each other. Data are presented as mean; sFAZ = superficial retinal plexus area; dFAZ = deep retinal plexus area; BCVA = best corrected visual acuity.

Statistically significant and negative correlations were found between ERM severity (Groups 1 and 2) and baseline sFAZ (r = -0.52, p = 0.004), dFAZ (r = -0.43, p = 0.027), as well as BCVA (r = -0.42, p = 0.028, see Fig 3). Further, ERM severity correlated statistically significantly with baseline CST (r = 0.58, p<0.001), ΔCST (r = 0.39, p = 0.028).

All other variables, the difference from baseline to month 3 in superficial FAZ area (ΔsFAZ), the difference from baseline to month 3 in deep FAZ area (ΔdFAZ), baseline VD1 and VD2-5, the difference from baseline to month 3 in VD1 (ΔVD1), the difference from baseline to month 3 in VD2-5 (ΔVD2-5), the difference from baseline to month 3 in BCVA (ΔBCVA), peeling size, and age, showed non-significant correlations (all p>0.05) with ERM severity.

A subgroup analysis of group 1 and 2, correlating best corrected visual acuity (BCVA) and vascular parameters (sFAZ, dFAZ, VD1, VD2-5), revealed non-significant correlations in both groups over time, except for VD1-BCVA at baseline (r = -0.7, p = 0.005) in group 1 and sFAZ-BCVA at week one (r = 0.62, p = 0.04) in group 2.

The SSOCTA-derived vascular variables showed statistically significant correlations between ΔsFAZ and ΔVD2-5 (r = 0.37, p = 0.04), but not ΔVD1 (r = 0.28, p = 0.082). Further, statistically significant correlations were found between ΔdFAZ and ΔVD1 (r = 0.38, p = 0.034), as well as ΔVD2-5 (r = 0.44, p = 0.014). Point-biserial correlations between ΔsFAZ (r = 0.36, p = 0.47), as well as ΔdFAZ (r = 0.52, p = 0.003) with the presence of intraretinal cysts were statistically significant.

The correlation of ΔsFAZ and ΔdFAZ showed non-significant correlations (all p > 0.05) with baseline BCVA (r = -0.15 and r = -0.06, respectively), ΔBCVA (r = -0.24 and r = -0.29, respectively), age (r = 0.22 and r = -0.12, respectively), peeling size (r = 0.16 and r = -0.01, respectively), and ΔCST (r = 0.02 and r = 0.005, respectively). ΔVD1 as well as ΔVD2-5 (r = 0.5 and r = 0.39, respectively; both p> 0.05) showed non-significant correlations with ΔBCVA.

Point-biserial correlations between pseudophakia and superficial FAZ (r = 0.12, p = 0.35) as well as deep FAZ (r = 0.26, p = 0.08) were not statistically significant. Correlations between pseudophakia and BCVA were not statistically significant (r = -0.34, p = 0.76) at baseline or 3 months after surgical intervention (r = -0.25, p = 0.18). However, a statistically significant correlation was found between ΔBCVA and ΔCST (r = -0.47, p = 0.013). No correlations for subretinal fluid were calculated as only three patients presented with subretinal fluid at baseline.

## Discussion

With the availability of high-resolution SSOCTA technology, a non-invasive imaging tool has become available raising hopes for new biomarkers that can predict the visual outcome and therapeutic response in retinal and choroidal disease, including idiopathic ERM. The recent literature on OCT/OCTA and ERM highlights changes during the disease process and after surgical intervention, with vascular changes occurring predominantly in the superficial and deep retinal plexus. With this study, we tried to take current knowledge a step forward by describing the natural course of SSOCTA variables in patients following ERM and ILM peeling and further by correlating SSOCTA variables with ERM severity based on OCT staging, functional and morphologic features. This is of interest as idiopathic ERM is a common retinal disease that critically affects visual function [4] and prognostic biomarkers need to be identified to better predict visual outcome and plan surgical intervention.

This study shows a clear influence of ERM severity derived from ERM stage on pre-surgical treatment factors. Statistically significant correlations were found between ERM severity (Groups 1 and 2) and baseline sFAZ, dFAZ as well as BCVA, CST and ΔCST. Further, at baseline as well as at the 3-month follow-up superficial and deep retinal FAZ areas decreased with ERM severity increase. Foveal VD increased with ERM severity at baseline. This effect diminished 3 months after ERM and ILM peeling. Although currently clinically not relevant, the association between SSOCTA factors and ERM stage may aide in the process of taking ERM severity assessment to a more uniform and automated analysis in future.

Statistically significant differences between ERM severity groups were found for sFAZ and dFAZ, both at baseline and the 3-month follow-up. The differences between groups in foveal and parafoveal VD were not statistically significant. Also, a subgroup analysis of groups 1 and 2, correlating BCVA and vascular parameters (sFAZ, dFAZ, VD1, VD2-5), revealed non-significant correlations in both groups over time, except for VD1-BCVA at baseline (r = -0.7, p = 0.005) in group 1 and sFAZ-BCVA at week one (r = 0.62, p = 0.04) in group 2. We consider these findings to be by chance, as out of 40 correlations performed only two were statistically significant and as all other time points showed no statistically significant correlations.

This study revealed statistically significant correlations between ΔsFAZ and ΔVD2-5, and between ΔdFAZ and ΔVD1 as well as ΔVD2-5. All variables were positively correlated, indicating that the bigger the change in FAZ area, the bigger is the change in VD during the first 3 months. This shows, that FAZ and VD in patients with ERM are variables dependent on each other; a finding interesting for future studies.

Age, peeling size, pseudophakia, and ΔCST were not statistically significantly correlated with ΔsFAZ and ΔdFAZ, but the presence of intraretinal cysts was statistically significantly correlated. Regarding BCVA, no statistically significant correlations of ΔBCVA with the SSOCTA variables ΔsFAZ, ΔdFAZ, ΔVD1 or ΔVD2-5 could be found during the first 3 months after surgical intervention. The correlation with ΔCST, however, was statistically significant.

These findings are of special interest as up until now the ERM severity or stage have not been considered in studies using OCTA reporting FAZ or VD. This may explain the variation in earlier results, which we refer to in the following.

Our findings contradict previous studies on the correlation between sFAZ area and BCVA that showed the smaller the preoperative FAZ area, the poorer was the postoperative BCVA [23,24]. They also contradict a recent retrospective case series that showed a statistically significant correlation between FAZ area and visual outcome 6 months after surgical intervention [28]. However, the approach used was slightly different from ours as the interocular differences in FAZ area and parafoveal VD of the superficial and deep plexus were used for the correlation analysis, which indicated that greater interocular differences were associated with worse postoperative BCVA. In an exploratory approach, we could not confirm these findings in our patient cohort. Correlating both mean sFAZ and dFAZ area difference to control eyes with 3-month BCVA showed non-significant and weak correlations (r = 0.04 and r = -0.14, respectively; each p> 0.05). Possible reasons for the discrepancy could be the ERM stage, 3-month difference in follow-up or the different devices used with higher resolutions available in SSOCTA technology [22]. A recent study revealed that the biggest change in the hemodynamic OCTA variables occurs during the first month after treatment [11]. This indicates that the difference between our results and the finding of statistically significant correlations between hemodynamic SDOCTA variables and function after ERM and ILM peeling [28] are due ERM stage or the OCTA technologies used rather than the follow-up period.

Common findings reported in various studies are smaller baseline FAZ area and fluctuant floveal, para- and perifoveal VD of both the superficial and deep retinal plexus compared with healthy contralateral control eyes [10,11,23,24,28–30]. Inconsistent results after 6 months of follow-up show no change in sFAZ and dFAZ [31], or significantly increased sFAZ [32] as well as statistically significantly increased parafoveal and slightly increased perifoveal perfusion density compared with baseline [11]. This accords with our results, showing smaller baseline sFAZ and dFAZ area compared with healthy contralateral control eyes. At the 3-month follow-up sFAZ and dFAZ area reached baseline values again after an initial decrease following surgery. However, at baseline our study showed higher foveal VD values (VD1) and identical parafoveal VD values (VD2-5) compared with healthy contralateral control eyes. VD ETDRS 1 showed an initial increase and returned to near-baseline values after 3 months. VD ETDRS 2–5 decreased by 4.6% until month one after surgery and remained stable thereafter.

The above-summarized findings are not surprising as previous studies showed retinal vessel displacement and macular constriction in ERM eyes, which can be attributed to a centripetal and tangential traction during ERM and ILM peeling compressing or curling retinal vessels in one place and straightening vessels in other areas of the retina [33], explaining variable results. ERM and ILM peeling releases these forces and the retinal plexuses, both the superficial [34] and deep may return towards their physiological position. The physiological location of the superficial vascular plexus is within the nerve fiber and ganglion cell layers, and the deep vascular plexus is located in the inner nuclear and outer plexiform layers [35]. A previously reported thickness reduction of the ganglion cell layer after ERM and ILM peeling may further aid in the restoration of the superficial retinal vascular plexus [36]. The inhomogeneity in foveal and parafoveal VD difference of the ERM eye compared to the contralateral control eye may further be explained by the fact, that foveal VD compares one ETDRS subfield between eyes, whereas parafoveal VD compares the mean of 4 ETDRS subfields. Consequently, the mathematical average of areas with higher VD and areas with lower VD may compare to the VD of the contralateral control eye. An assumption to be confirmed in a future study.

Preliminary results indicate that mechanical forces might affect the blood flow in the DCP more than in the SCP, with the flow in both improving one month after ERM peeling [37]. This finding could be of interest as the deep capillary plexus is located within the INL and OPL. The OPL is co-localized with the lowest oxygen tension in the retina [38]. Studies reporting VD alterations in the deep retinal plexus could lend support to the suggestion mechanical forces have more effect on the flow of the DCP. Consequently, this data may present an argument for performing ERM + ILM peeling early in the disease process as statistically significant differences between ERM severity groups were found not only for visual acuity but also for the vascular variables superficial and deep capillary plexus FAZ as well as foveal VD (VD1). Following the above-mentioned oxygen hypothesis, a longer wait for surgical intervention would result in long-lasting critical diatomic oxygen shortage. Consequently, a timely intervention in early ERM stages may prevent undersupply. This, however, is a tough question which needs to be answered in a longitudinal study as the wait for a clinically indicated surgical intervention should not be intentionally deferred.

For VD interpretations caution is advised as different ways of computation are used. The most basic definition of vessel or vascular density is the ratio of pixels representing vessels over the total area investigated. Image processing before VD calculation varies greatly and is sometimes not known to the user. Also previously shown, retinal perfusion indices in different-sized OCTA volumes are not interchangeable [39].

The associations found between ΔBCVA and ΔCST indicate that the bigger the effect of ERM + ILM peeling on CST, the great the gain in BCVA.

Some limitations have to be considered. As consecutive patients were included in this study, the distribution among ERM stages is very heterogeneous, therefore patients were grouped based on ERM severity. Future studies will have to evaluate the influence of each individual ERM stage in more detail. Various ERM studies report a 6-month follow-up period, which we omitted as statistically significant morphological changes have been shown to occur within month one [11]. Baseline BCVA may be biased by the presence of cataract in the study eye, a finding frequent in ERM eyes. However, we consider this bias to be negligible as an image quality score below 40 was considered an exclusion criterion. Further, correlations between pseudophakia and BCVA were not statistically significant (r = -0.34, p = 0.76) at baseline or 3 months after surgical intervention (r = -0.25, p = 0.18).

The influence of axial eye length on OCT/A measurements could be omitted as we excluded patients exceeding +/- 3 diopters of spherical equivalent. Although, we could not show statistically significant correlations between pseudophakia and BCVA, the influence of a combined procedure on visual acuity cannot be waived. To ensure comparability in future studies a consensus on automated VD calculations or a manual approach would be helpful. Also, averaging of multiple OCTA images [38] could help refine OCTA-derived metrics. Furthermore, the intermediate retinal plexus [40] was not considered in this study, as the device used does not differentiate between all three retinal plexuses.

In conclusion, we could not prove that the hemodynamic SSOCTA variables sFAZ and dFAZ area, as well as foveal and parafoveal VD are of prognostic value for post-surgical treatment BCVA. Despite the obvious advantages OCTA-derived variables would have in the prognosis of BCVA, the clinical usefulness of OCTA in eyes with idiopathic ERM remains questionable. However, this study clearly shows ERM severity or staging should be taken into account when investigating patients with idiopathic ERM using SSOCTA.
